# Cyclic peptide *CRRETAWAC* attenuates fibronectin‐induced cytokine secretion of human airway smooth muscle cells by inhibiting FAK and p38 MAPK

**DOI:** 10.1111/jcmm.13174

**Published:** 2017-04-12

**Authors:** Mengdi Chu, Jiani Ji, Wenhao Cao, Huojun Zhang, Dan Meng, Bangruan Xie, Shuyun Xu

**Affiliations:** ^1^ Department of Respiratory and Critical Care Medicine Tongji Hospital Tongji Medical College Huazhong University of Science and Technology Hubei China; ^2^ Ningbo Medical Treatment Center Lihuili Hospital Ningbo China; ^3^ Wuhan Medical Treatment Center Hubei China

**Keywords:** asthma, fibronectin, airway smooth muscle, integrin α5β1

## Abstract

α5β1 integrin is highly expressed in airway smooth muscle cells and mediate the adhesion of airway smooth muscle cells to fibronectin to regulate airway remodelling in asthma. This study aimed to investigate the effects of synthetic cyclic peptide *CRRETAWAC* on fibronectin‐induced cytokine secretion of airway smooth muscle cells and the underlying mechanism. Human airway smooth muscle cells were isolated and treated with fibronectin, IL‐13, *CRRETAWAC* peptide, α5β1 integrin‐blocking antibody, FAK inhibitor or p38 MAPK inhibitor. The transcription and secretion of eotaxin‐1 and RANTES were detected by real‐time PCR and ELISA, respectively. The phosphorylation of FAK and MAPKs including p38, ERK1/2 and JNK1/2 was detected by Western blot analysis. The transcription and secretion of eotaxin‐1 and RANTES increased in airway smooth muscle cells cultured in fibronectin‐coated plates. However, α5β1 integrin‐blocking antibody, *CRRETAWAC* peptide, FAK inhibitor or p38 MAPK inhibitor significantly reduced mRNA levels and the secretion of eotaxin‐1 and RANTES in airway smooth muscle cells cultured in fibronectin‐coated plates. In addition, the phosphorylation of FAK and p38 MAPK was significantly increased in airway smooth muscle cells cultured in fibronectin‐coated plates compared to the cells cultured in uncoated plates and was significantly reduced in airway smooth muscle cells treated with *CRRETAWAC* peptide. Fibronectin induces cytokine synthesis and secretion of airway smooth muscle cells. Peptide *CRRETAWAC* antagonizes fibronectin‐induced cytokine synthesis and secretion of airway smooth muscle cells *via* the inhibition of FAK and p38 MAPK, and is a potential agent for the therapy of asthma.

## Introduction

Asthma is characterized by reversible airway obstruction and airway hyper‐responsiveness that is associated with airway inflammation and airway remodelling 1, 2. Two prominent pathological features of asthma are the increase of airway smooth muscle (ASM) mass and the deposition of extracellular matrix (ECM) proteins, which contribute to the development of airway inflammation and remodelling. The deposition of increased ECM proteins such as fibronectin and collagen in ASM layer has been observed in asthma 3, 4. Evidence suggests that increased ECM deposition could induce ASM phenotype switching from the contractile phenotype to the proliferative phenotype, accompanied by increased expression of cell adhesion receptors and costimulatory molecules as well as enhanced secretion of cytokines and chemokines that activate eosinophils such as eotaxin, RANTES and GM‐CSF 5, 6, 7.

Integrins are a group of transmembrane heterodimeric proteins that mediate cell–cell and cell–ECM interactions. Eighteen α‐subunits and eight β‐subunits dimerize non‐covalently to form at least 24 different integrin heterodimers with specific tissue distribution 8. The extracellular domain of integrin recognizes short peptide sequence Arg‐Gly‐Asp (RGD), which presents in ECM proteins such as fibronectin, collagen I, laminin and vitronectin 9, 10. The intracellular domain of integrin is involved in the formation of focal adhesion complexes to mediate downstream signalling events 11. Blocking the ECM‐binding domain of integrin is a valid approach for the inhibition of integrin signalling 12. Different integrin antagonists for preventing ligand binding have currently being investigated such as antibodies, (cyclic) peptides and small molecules 12, 13.

Integrins are essential for mediating the interaction of airway smooth muscle cells (ASMCs) to the surrounding ECM 12. ASMCs express integrin α1‐7, α9, αV, β1, β3 and β5, and particularly highly express α5β1 13. Integrin α5β1 is the major fibronectin‐binding integrin and a series of *in vitro* studies have demonstrated that integrin α5β1 mediates the depression of contractility and the enhancement of ASM proliferation and cytokine secretion induced by fibronectin, which are inhibited by α5β1 function‐blocking antibodies and RGD‐blocking peptide Arg‐Gly‐Asp‐Ser (RGDS) 5, 6, 11, 14, 15, 16. In particular, RGDS peptide attenuated allergen‐induced ASM hyperplasia and hypercontractility *in vivo* 16. Therefore, the antagonists of integrin α5β1 have therapeutic potential as anti‐inflammation and antiremodelling agents for asthma. The integrin/ECM‐blocking peptide RGDS has been demonstrated to be an attractive therapeutic agent in asthma. However, RGDS peptide is not specific for integrin α5β1. Several ECM components such as collagen I, fibronectin and laminin all contain the RGD‐binding motif 9, 10. The synthetic cyclic peptide H‐Cys*‐Arg‐Arg‐Glu‐Thr‐Ala‐Trp‐Ala‐Cys*‐H (*CRRETAWAC*) is a highly specific ligand for α5β1 integrin derived from phase display 17. This peptide has been shown to specifically block integrin α5β1‐mediated cell attachment to fibronectin 18, 19, 20. However, it remains unknown whether *CRRETAWAC* could antagonize the changes of ASMCs phenotype and function induced by fibronectin during asthma.

In this study, we aimed to investigate the potential of *CRRETAWAC* peptide to inhibit IL‐13‐dependent cytokine production of human ASMCs induced by fibronectin and explore the underlying mechanism.

## Materials and Methods

### Reagents

Peptides GACRRETAWACGA (CRRETAWAC), GACRRETADACGA (CCRETADAC) and GCRGDSPCG (cyclic‐RGD) were purchased from GL Biochem Ltd (Shanghai, China). Peptides were cyclized by oxidation as described previously 18. Recombinant human IL‐13 was purchased from Peprotech Inc (Rocky Hill, NJ, USA).

### Isolation and culture of human ASMCs

Macroscopically, normal human lungs were obtained in accordance with procedures approved by the Tongji Hospital's Research Ethics Committee from six patients without asthma (mean age 59 years; range 50–70 years; three male, three female) undergoing lung partial resection for carcinomas. ASMCs were cultured from the main or lobar bronchus as described previously 21. ASMCs were characterized by fluorescent immunocytochemical staining of α‐actin. The purity of ASMCs at passage 3 was more than 95%. Cells at passage 3–6 were used in all experiments.

Culture plates were coated with human fibronectin (Hyclone, USA, 10 μg/ml diluted in PBS) overnight at 37°C, and the unbound fibronectin was removed by aspiration and washing with PBS. Unoccupied protein‐binding sites were blocked by incubation with 0.1% bovine serum albumin for 30 min. at room temperature. ASMCs in Dulbecco's modified Eagle's medium (DMEM) containing 5% FBS were seeded (10,000 cells/plate) in plates with or without fibronectin pre‐coating and cultured for 24 hrs, and then, the medium was replaced with FBS‐free DMEM and cultured for another 24 hrs. ASMCs were then pre‐treated for 30 min. with anti‐integrin α5 monoclonal antibodies or corresponding isotype‐matched control antibodies (R&D systems, USA), or the peptides. After ASMCs were stimulated with IL‐13 for 6 hrs, eotaxin‐1 and RANTES mRNA in the cell extracts were analysed by real‐time PCR. After ASMCs were stimulated with IL‐13 for 24 hrs, eotaxin‐1 and RANTES protein in culture supernatants were analysed by ELISA. The protein in the cell lysates was analysed by Western blot.

### Real‐time PCR

Total RNA was isolated from AMSCs using TRIzol (Invitrogen). cDNA was synthesized by reverse transcription using RT kit (TaKaRa, Japan) following the manufacturer's protocol. Real‐time PCR was performed using the following primers synthesized by Sango (Shanghai, China): Eotaxin‐1 gaaggtctccgcagcact (forward) and acttcttcttggggtcgg (reverse); RANTES ccctcgctgtcatcctca (forward) and cacttggcggttctttcg (reverse); β‐actin ctgggacgacatggagaaaa (forward) and aaggaaggctggaagagtgc (reverse). Data were analysed with the 2^−▵▵Ct^ method and presented as the fold change in mRNA expression normalized to β‐actin: ▵▵Ct=(Ct_target gene_‐Ct_action_)_treatment_‐(Ct_target gene_‐Ct _action_)_control_.

### Western blot analysis

ASMCs were collected and total protein was isolated from the cells and total proteins quantitated by BSA method, separated by 10% SDS–PAGE and transferred to PVDF membrane (Millipore, Billerica, MA**,** USA). Next, the membrane was blocked in 5% powdered milk in TBST (Tris–HCl buffer containing Tween‐20) for 1 hr at room temperature, and then incubated with specific antibody for FAK, p38, ERK and p‐JNK or β‐actin (Cell Signaling, Danvers, MA, USA) at 4°C overnight. The membrane was washed with TBST three times, and then incubated with secondary antibody (Cell Signaling, Danvers, MA, USA) for 1 hr at room temperature. The membrane was washed with TBST three times and developed using enhanced chemiluminescence reagents. Bands were quantified with Image Lab system (Bio‐Rad Laboratories, USA) and are presented as the relative ratio to GAPDH.

### Elisa

Cytokines and chemokines in culture supernatants were determined by sandwich ELISA performed with the kits (PeproTech, USA). Recombinant human eotaxin‐1 standards and samples were added to the plates which were coated with anti‐eotaxin‐1 antibody, and then incubated overnight at 4°C. Biotinylated detection antibodies were added to the plates, and incubated at room temperature for 2 hrs, followed by incubation with peroxidase‐labelled streptavidin and TMB substrate.

### Statistical analysis

Data were presented as means ± S.D. and analysed using one‐way or two‐way ANOVA followed by Turkey's test. GraphPad software was used for all statistical analysis. Statistical significance was determined as *P* < 0.05.

## Results

### Integrin α5β1 contributed to IL‐13‐dependent and fibronectin‐induced cytokine production by ASMCs

**Figure 1:**
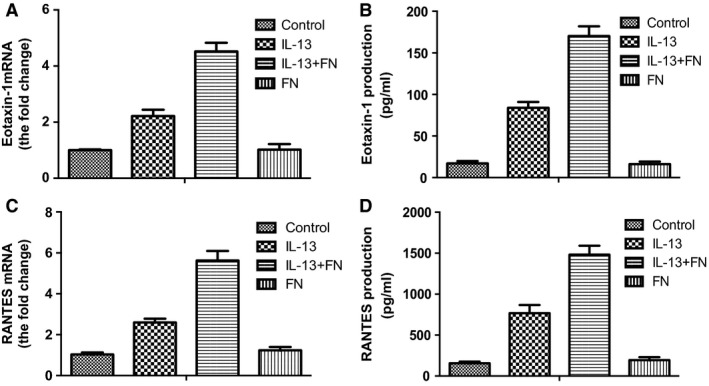
Fibronectin induced cytokine production by ASMCs. AMSCs were cultured in uncoated plates or plates pre‐coated with fibronectin and treated with or without IL‐13 (20 ng/ml). (**A**) The mRNA level of eotaxin‐1 in ASMCs was detected by real‐time PCR. (**B**) The concentration of eotaxin‐1 in culture medium of ASMCs was detected by ELISA. (**C**) The mRNA level of RANTES in ASMCs was detected by real‐time PCR. (**D**) The concentration of RANTES in culture medium of ASMCs was detected by ELISA. All data were presented as means ± S.D. (*n *=* *3). ASMCs: airway smooth muscle cells. ASMCs: airway smooth muscle cells.

First, we determined whether the cytokine production of ASMCs increased after treatment with IL‐13 and fibronectin. IL‐13 (20 ng/ml) increased the expression of eotaxin‐1 and RANTES mRNA levels as well as the secretion of eotaxin‐1 and RANTES of ASMCs compared to unstimulated cells (*P*<0.05; Fig. 1). For cells cultured in plates pre‐coated with fibronectin, IL‐13 stimulated higher expression of eotaxin‐1 and RANTES mRNA levels as well as the secretion of eotaxin‐1 and RANTES compared with cells cultured in uncoated plates (*P*<0.05; Fig. 1). Furthermore, fibronectin could not increase the expression and secretion of eotaxin‐1 and RANTES in ASMCs without IL‐13 stimulation (*P*>0.05; Fig. 1).

**Figure 2:**
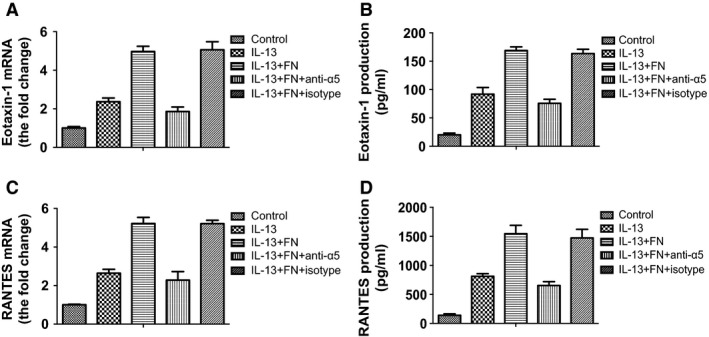
Integrin α5β1 antibody inhibited fibronectin‐induced cytokine production by ASMCs. AMSCs were cultured in uncoated plates or plates pre‐coated with fibronectin and treated with or without IL‐13 (20 ng/ml), or with integrin α5β1 antibody or isotype control antibody. (**A**) The mRNA level of eotaxin‐1 in ASMCs was detected by real‐time PCR. (**B**) The concentration of eotaxin‐1 in culture medium of ASMCs was detected by ELISA. (**C**) The mRNA level of RANTES in ASMCs was detected by real‐time PCR. (**D**) The concentration of RANTES in culture medium of ASMCs was detected by ELISA. All data were presented as means ± S.D. (*n *=* *3). ASMCs: airway smooth muscle cells.

Next, we wondered whether integrin α5β1 is involved in IL‐13‐dependent fibronectin‐induced cytokine production in ASMCs. We used integrin α5β1 functional antibody to specifically block the binding of fibronectin to integrin α5β1 expressed on ASMCs. Compared with isotype control antibody, α5β1 integrin‐blocking antibody effectively prevented the enhancement of IL‐13‐dependent and fibronectin‐induced eotaxin‐1 and RANTES mRNA expression (*P*<0.05; Fig 2A and C) and secretion (*P*<0.05; Fig 2B and D). These data suggest that integrin α5β1 contributes to IL‐13‐dependent and fibronectin‐induced cytokine production by ASMCs.

### *CRRETAWAC* reduced the enhancement of IL‐13‐dependent cytokine production of human ASMC induced by fibronectin

**Figure 3:**
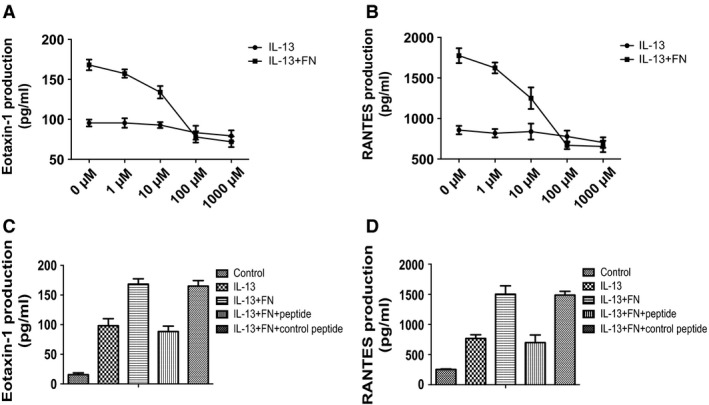
*CRRETAWAC* peptide inhibited fibronectin‐induced cytokine production by ASMCs. AMSCs were cultured in uncoated plates or plates pre‐coated with fibronectin and treated with IL‐13 (20 ng/ml) in the presence of different concentration of *CRRETAWAC* peptide (from 0 to 1,000 μM). (**A**) The concentration of eotaxin‐1 in culture medium of ASMCs was detected by ELISA. (**B**) The concentration of RANTES in culture medium of ASMCs was detected by ELISA. AMSCs were cultured in uncoated plates or plates pre‐coated with fibronectin and treated with IL‐13 (20 ng/ml), or *CRRETAWAC* peptide or control peptide (100 μM). (**C**) The concentration of eotaxin‐1 in culture medium of ASMCs was detected by ELISA. (**D**) The concentration of RANTES in culture medium of ASMCs was detected by ELISA. All data were presented as means ± S.D. (*n *=* *3). ASMCs: airway smooth muscle cells. ASMCs: airway smooth muscle cells.

As *CRRETAWAC* peptide could specially block the binding of Integrin α5β1 to fibronectin, we examined whether it inhibits IL‐13‐dependent and fibronectin‐induced cytokine production by ASMCs. The results showed that *CRRETAWAC* dose dependently (from 0 to 100 μM) decreased IL‐13‐dependent and fibronectin‐induced secretion of eotaxin and RANTES by ASMCs. However, there were no significantly differences between the dose of 100 μM and 1000 μM of peptide (Fig. 3A and B). Therefore, we chose 100 μM of peptide for further experiments.

We used *CAWAC* peptide as the control peptide which has a different amino acid in the key location. Compared to *CAWAC* peptide, *CRRETAWAC* significantly inhibited IL‐13‐dependent and fibronectin‐induced eotaxin‐1 and RANTES mRNA expression and secretion in ASMCs (Fig. 3C and D).

### FAK regulated cytokine production of ASMCs induced by fibronectin

**Figure 4:**
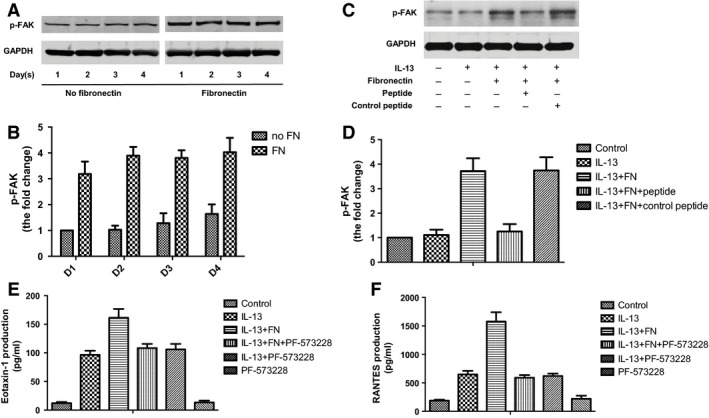
FAK regulated cytokine production of ASMCs induced by fibronectin. (**A**) ASMCs were cultured in uncoated plates or plates pre‐coated with fibronectin for one to 4 days. Phosphorylated FAK was detected by Western blot analysis. GAPDH was loading control. (**B**) Densitometry analysis of p‐FAK levels shown in A. (**C**) ASMCs were cultured in uncoated plates or plates pre‐coated with fibronectin and treated with IL‐13 (20 ng/ml), *CRRETAWAC* peptide or control peptide (100 μM). Phosphorylated FAK was detected by Western blot analysis. GAPDH was loading control. (**D**) Densitometry analysis of p‐FAK levels shown in C. E and F. ASMCs were cultured in uncoated plates or plates pre‐coated with fibronectin and treated with IL‐13 (20 ng/ml) or PF‐573228 (100 nM). The concentration of eotaxin‐1 (**E**) and RANTES (**F**) in culture medium of ASMCs was detected by ELISA. All data were presented as means ± S.D. (*n *=* *3). ASMCs: airway smooth muscle cells.

To understand the signalling mechanism by which fibronectin stimulated eotaxin and RANTES production by ASMCs, we focused on FAK activation. Western blot analysis showed that the phosphorylation of FAK increased in ASMCs cultured in fibronectin‐coated plates compared with ASMCs cultured in uncoated plates (Fig. 4A and B).

Next, we investigated the effect of *CRRETAWAC* peptide on FAK phosphorylation induced by fibronectin. Western blot analysis showed that *CRRETAWAC* significantly inhibited FAK phosphorylation induced by fibronectin and IL‐13 compared with control peptide (Fig. 4C and D). IL‐13 alone did not affect FAK phosphorylation.

To investigate whether FAK is required for eotaxin and RANTES secretion induced by fibronectin, we used specific FAK inhibitor PF‐573228 to treat ASMCs. We found that PF‐573228 antagonized enhanced secretion of eotaxin and RANTES induced by fibronectin and IL‐13 (Fig. 4E and F). PF‐573228 did not affect eotaxin and RANTES secretion induced by IL‐13 alone (Fig. 4E and F). Collectively, these results indicate that FAK regulates cytokine production of ASMCs induced by fibronectin.

### p38 MAPK signalling regulated cytokine production of ASMCs induced by fibronectin

**Figure 5:**
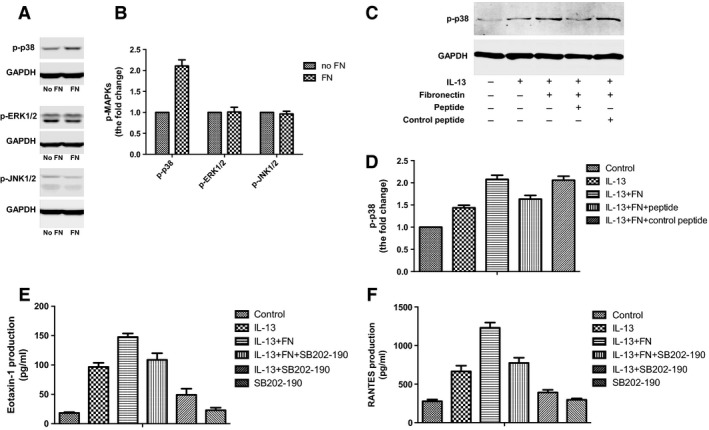
p38 MAPK regulated cytokine production of ASMCs induced by fibronectin. (**A**) ASMCs were cultured in uncoated plates or plates pre‐coated with fibronectin for 1 day. Phosphorylated p38 MAPK was detected by Western blot analysis. GAPDH was loading control. (**B**) Densitometry analysis of p‐p38 MAPK levels shown in A. (**C**) ASMCs were cultured in uncoated plates or plates pre‐coated with fibronectin and treated with IL‐13 (20 ng/ml), *CRRETAWAC* peptide or control peptide (100 μM). Phosphorylated p38 MAPK was detected by Western blot analysis. GAPDH was loading control. (**D**) Densitometry analysis of p‐p38 MAPK levels shown in C. E and F. ASMCs were cultured in uncoated plates or plates pre‐coated with fibronectin and treated with IL‐13 (20 ng/ml) or SB202‐190 (10 μM). The concentration of eotaxin‐1 (**E**) and RANTES (**F**) in culture medium of ASMCs was detected by ELISA. All data were presented as means ± S.D. (*n *=* *3). ASMCs: airway smooth muscle cells.

Furthermore, we detected the status of MAPK signalling in ASMCs treated with fibronectin. Western blot analysis showed that fibronectin activated p38 phosphorylation but had no significant effects on ERK or JNK phosphorylation in ASMCs (Fig. 5A and B).

Next, we examined the effect of *CRRETAWAC* peptide on p38 MAPK phosphorylation induced by fibronectin. Western blot analysis showed that *CRRETAWAC* significantly inhibited p38 phosphorylation induced by fibronectin and IL‐13 compared with control peptide (Fig. 5C and D).

To investigate whether p38 MAPK is essential for eotaxin and RANTES secretion induced by fibronectin, we employed SB202‐190 as the inhibitor of p38 MAPK to treat ASMCs. We found that SB202‐190 antagonized enhanced secretion of eotaxin and RANTES induced by fibronectin and IL‐13 (Fig. 5E and F). Taken together, these data suggest that p38 MAPK regulates cytokine production of ASMCs induced by fibronectin.

## Discussion

In present study, we provided the first evidence that integrin α5β1 contributed to IL‐13‐dependent eotaxin and RANTES production and secretion by human ASMCs under stimulation by fibronectin. Furthermore, we demonstrated that synthetic cyclic peptide *CRRETAWAC* specifically blocked the binding of integrin α5β1 and fibronectin, reduced IL‐13‐dependent and fibronectin‐induced eotaxin and RANTES production and secretion by human ASMCs, and inhibited the activation of FAK and p38 MAPK.

Chronic airway inflammation and airway remodelling are important characteristics of asthma 1. ASMCs are crucially implicated in airway inflammation and remodelling 3. Under pathological stimuli, ASMCs produce abundant cytokines, chemokines, cell adhesion molecules and ECM proteins *via* autocrine and paracrine. These factors promote the proliferation and differentiation of ASMCs, forming a positive feedback loop 22. Furthermore, these factors enhance the recruitment and activation of eosinophils to modulate airway inflammation and remodelling 23. Eotaxin and RANTES are eosinophil‐activating cytokines, and contribute to airway inflammatory events 5. Therefore, in this study, we chose these two cytokines to investigate the effects of *CRRETAWAC* peptide to inhibit cytokine production of human ASMCs induced by fibronectin. We found that the synthesis and secretion of eotaxin and RANTES by ASMCs were significantly induced by fibronectin, but they were inhibited by *CRRETAWAC* peptide. These data suggest that *CRRETAWAC* peptide could antagonize inflammatory reactions in ASMCs and thus has beneficial effects on asthma.

Integrin α5β1 is universally expressed in ASMCs and is a major fibronectin receptor that mediates the depression of contractility and the enhancement of ASMCs proliferation and cytokine secretion induced by fibronectin 5, 11, 12, 13, 14. The attachment of ASMCs to fibronectin could be inhibited by blocking of α5 integrin subunit. RGDS peptide *in vivo* attenuated allergen‐induced ASMCs hyperplasia and hypercontractility 16. In addition, platelet‐derived growth factor‐BB‐stimulated proliferation of ASMCs was prevented by integrin α5β1‐blocking antibody 14. Furthermore, blocking α5 integrin abolished the enhancement of IL‐1β‐dependent eotaxin release induced by fibronectin 11. In this study, our data showed that fibronectin enhanced IL‐13‐dependent eotaxin and RANTES production by human ASMCs, which was inhibited by integrin α5β1 functional antibody. These results are consistent with previous findings that α5β1 was required for fibronectin‐enhanced IL‐13‐dependent eotaxin release by ASMCs from patients with asthma 5. These findings reveal that integrin α5β1 is a therapeutic target in asthma.

Recently, signalling mechanism downstream of integrin α5β1 becomes to be explored. The binding of integrin α5β1 to ECM promotes the recruitment and phosphorylation of FAK and other kinases 24. It was shown that integrin α5β1 interacted with ECM and activated ERK1 and ERK2 MAPKs, leading to osteoblast differentiation and skeletal development 25, 26. However, in this study, we found that fibronectin activated the phosphorylation of FAK and p38 MAPK but not the phosphorylation of ERK or JNK MAPKs in ASMCs. Furthermore, *CRRETAWAC* peptide inhibited fibronectin‐induced activation of FAK and p38 MAPK in ASMCs. These data suggest that in ASMCs, the binding of integrin α5β1 to ECM component fibronectin could promote the activation of FAK and p38 MAPK to mediate inflammatory and phenotypic changes of ASMCs. Further studies are needed to understand how FAK and p38 MAPK signalling pathways regulate the transcription and secretion of cytokines by ASMCs.

Due to therapeutic potential of the antagonists of integrin α5β1 as anti‐inflammation and antiremodelling agents for asthma, significant effects have been taken to develop different types of integrin α5β1 antagonists in the past several years. Among them, integrin/ECM‐blocking peptide RGDS has gained most attention because it has been shown to be effective to block integrin α5β1. However, RGD motif is present in many types of ECM proteins and binds a large number of integrins, including αIIbβ3 on the platelets as well as αvβ3 and α5β1 on endothelial cells 10. Therefore, specific integrin α5β1 antagonists are needed. The synthetic cyclic peptide *CRRETAWAC* is derived from a phage display library with a high affinity for human integrin α5β1, and specifically blocks integrin α5β1‐mediated cell attachment to fibronectin 17, 18, 19, 20. It is still unknown whether *CRRETAWAC* peptide could antagonize the phenotype changes of ASMCs induced by fibronectin. In this study, for the first time, our data demonstrate that in human ASMCs, *CRRETAWAC* peptide specifically and potently blocked the interaction of integrin α5β1 with fibronectin, inhibited fibronectin‐induced transcription and secretion of eotaxin and RANTES and abolished the activation of FAK and p38 MAPK pathways. It remains to be determined the *in vivo* efficacy, specificity and safety of *CRRETAWAC* peptide for airway inflammation and remodelling. Our next step is to administer *CRRETAWAC* peptide in animal model of asthma and investigate the outcome.

In conclusion, fibronectin induces cytokine synthesis and secretion of ASMCs in a manner dependent on α5β1 integrin and the activation of FAK and p38 MAPK. *CRRETAWAC* peptide antagonizes fibronectin‐induced cytokine synthesis and secretion of ASMCs, at least partially *via* the inhibition of FAK and p38 MAPK. Therefore, *CRRETAWAC* peptide is a potential agent for the therapy of asthma.

## Conflict of interest

The authors declare that they have no conflict of interest.
